# Leukemia Cutis With Concomitant Seborrheic Keratosis as the Presenting Symptom of Chronic Lymphocytic Leukemia: A Case Report

**DOI:** 10.7759/cureus.82433

**Published:** 2025-04-17

**Authors:** Madison Drallmeier, Lillian Ranspach, George Elias

**Affiliations:** 1 Internal Medicine, Henry Ford Health System, Detroit, USA; 2 College of Osteopathic Medicine, Michigan State University, East Lansing, USA; 3 Obstetrics and Gynecology, Ascension St. John Hospital, Detroit, USA; 4 Family Medicine, Corewell Health, Farmington Hills, USA

**Keywords:** aleukemic leukemia cutis, chronic lymphocytic leukemia (cll), leukemia cutis, seborrheic keratosis, small lymphocytic lymphoma (sll)

## Abstract

Leukemia cutis is a term used to describe a dermatologic manifestation of hematologic malignancies. It represents a wide range of identifiable cutaneous lesions that result from the infiltration of neoplastic leukocytes in patients with leukemia or lymphoma. We report a case involving an initial solitary lesion without systemic symptoms, but a biopsy revealed a seborrheic keratosis in the epidermis with lymphoid infiltration in the dermis. Flow cytometry studies confirmed a B-cell chronic lymphocytic leukemia, and an additional investigation with fluorescence in situ hybridization demonstrated a chromosomal deletion in the long arm of chromosome 13 at position 14, indicating a favorable prognosis. The patient was referred to a hematologist, who determined that no pharmacologic treatment was necessary at that time. The patient continues to follow up for monitoring of his disease.

## Introduction

Leukemia cutis (LC) is the result of leukemic cells invading the epidermis, dermis, or hypodermis of the skin, and it manifests as a visible lesion. Although LC has been described in many hematologic cancers, it is rare for a lesion to be the presenting symptom of the disease, representing less than 10% of cases [1]. These lesions are often heterogeneous and may mimic more common and benign dermatologic findings. Additionally, they can develop in areas of previous infection sites, existing skin lesions, or prior areas of trauma [1]. Here, we report a rare case involving a middle-aged male patient with LC within a newly developed seborrheic keratosis as an initial manifestation of chronic lymphocytic leukemia (CLL).

## Case presentation

A Caucasian male in his fifties with a past medical history of hypertension, hyperlipidemia, and basal cell carcinoma of the face status post excision (approximately 10 years ago) without recurrence presented to his primary care physician with a concern about the evolution of a painless skin lesion. The lesion was located in his upper left pectoralis muscle region. The patient denied any associated pruritus, tenderness, bleeding, or exudate arising from the lesion. The patient's main concern was that the lesion appeared to grow over time. The dimensions were approximately 6 by 3 millimeters. The lesion was raised and loculated, demonstrating multicolored brown and tan areas. Differential diagnoses of the patient’s skin lesion were seborrheic keratosis, epidermal nevus, basal cell carcinoma, and squamous cell carcinoma. The decision was made to remove the suspicious skin lesion and perform a shave biopsy. 

At the time of presentation, the patient denied any systemic symptoms including fatigue, weight loss, fevers, chills, night sweats, or lymphadenopathy. On exam, the patient was afebrile without signs of lymphadenopathy and no other skin lesions besides the suspicious lesion described above. The patient’s most recent complete blood count (CBC) was one month prior to his presentation during his annual physical, which showed a borderline leukocytosis at 10.9 x 10^9^/L with a slight lymphocytosis of 5.5 x 10^9^/L and eosinophilia at 0.6 x 10^9^/L. His complete metabolic profile was largely unremarkable. 

**Figure 1:**
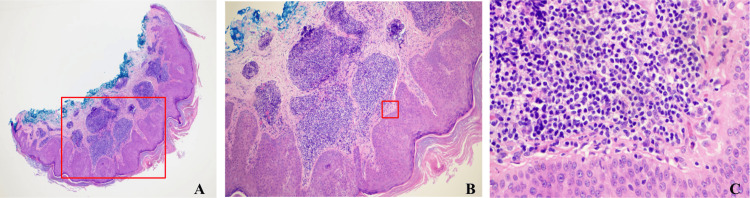
H&E stain of skin biopsy that demonstrated proliferation of epidermal keratinocytes consistent with seborrheic keratosis. Infiltration of small lymphocytes present in the dermis extending into the superficial dermis (A) H&E stain of skin biopsy at a magnification of 4X, where proliferation of epidermal keratinocytes can be appreciated. The red box demonstrates the area magnified in the next image. (B) H&E stain of skin biopsy at a magnification of  10X, where proliferation of epidermal keratinocytes can be appreciated as well as lymphocytic infiltration within the dermis. The red box demonstrates the area magnified in the next image. (C) H&E stain of skin biopsy at a magnification of 50X, where the small blue lymphocytes can be clearly seen in the dermis. H&E, hematoxylin and eosin.

**Figure 2:**
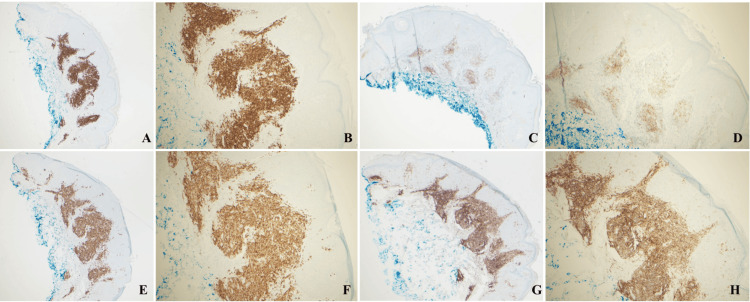
IHC of shave biopsy of patient's lesion with positive staining for CD20, CD5, CD23, and CD43 that is consistent with a diagnosis of B-cell CLL/SLL. A) IHC that demonstrates CD20-positive cells at 4X magnification. B) IHC that demonstrates CD20-positive cells at 10X magnification. C) IHC that demonstrates CD23-positive cells at 4X magnification. D) IHC that demonstrates CD23-positive cells at 10X magnification. E) IHC that demonstrates CD5-positive cells at 4X magnification. F) IHC that demonstrates CD5-positive cells at 10X magnification. G) IHC that demonstrates CD42-positive cells at 4X magnification. H) IHC that demonstrates CD42-positive cells at 10X magnification. IHC, immunohistochemistry; CLL, chronic lymphocytic leukemia; SLL, small lymphocytic lymphoma.

Biopsy of the lesion demonstrated a seborrheic keratosis in the epidermis with a dermal lymphocytic infiltrate that extends into the superficial dermis (Figure 1) and consisted of predominantly small B-cells (CD20+) that show co-expression of CD5 (dim), CD23, and CD43 (dim) (Figure 2).

This phenotype was consistent with a diagnosis of CLL/small lymphocytic lymphoma (SLL). The patient was referred to a hematologist for further diagnostic testing and workup. Fluorescence in situ hybridization (FISH) analysis of peripheral blood samples showed a chromosome 13q14 deletion. Flow cytometry analysis was also performed on blood samples and detected a population of monoclonal CD5+ B-cells comprising 32% of total cells. This immunophenotype can be seen in CLL/SLL but can also be present in other lymphoma subtypes. CD38 was not detected, indicating a favorable prognostic indicator in the setting of CLL/SLL. 

Ultimately, his hematologist deferred pharmacological treatment due to the indolence of his CLL/SLL. He was instructed that if he were to develop anemia, thrombocytopenia, severe lymphadenopathy or splenomegaly, and/or B symptoms, he should consider initiating therapy. The patient continues regular follow-up with his primary care physician and his hematologist. Fortunately, he has not had recurrence of LC around the prior site/scar, nor has he developed any additional concerning skin lesions. Although he has been noted to have an increase in his white count (34 x 10^9^/L), his other cell counts remain stable, and he denies systemic symptoms. Therefore, he does not need to start treatment and maintains close monitoring. 

## Discussion

LC describes the cutaneous manifestation of leukemia cells migrating from the bloodstream into the skin. It is a rare consequence of hematologic malignancies but has also been described in those with other blood diseases such as myelodysplastic syndromes or lymphoproliferative disorders (arising from both B- and T-cell lineages). One such blood cancer that LC is commonly associated with is the slow-growing B-cell CLL/SLL, which occurs in 4-20% of cases [1-3]. While LC usually arises after a diagnosis of systemic leukemia, approximately 10% of LC cases present as the initial symptom of the disease [1]. Lesions typically present as single or grouped, violaceous, erythematous, or reddish-brown in color and manifest as papules, plaques, nodules, or tumors [3,4]. They often arise on the trunk or extremities, but may also appear on the face, scalp, and genitalia. Interestingly, some studies have reported LC arising in sites of prior herpetic lesions, borrelia burgdorferi infections, and trauma [1,3,4]. Our patient presented one month after the development of a suspicious-appearing brown and tan raised lesion that arose on his chest in an area with no prior lesion or injury and without any systemic signs or symptoms that would indicate a leukemia diagnosis. Ultimately, presentation may be widely diverse and mimic other dermatologic conditions, which is why biopsy and histology examination are crucial in the diagnosis of LC [2]. 

Current guidelines in the diagnosis of CLL/SLL require that the cancer cells express the antigens CD19, CD20, and CD23 (markers of mature B-cells) as well as CD5 [5]. CD5 is generally present on T cells but is also known to be expressed on B1 cells, a subset of B cells that are responsible for producing antibodies and exhibit "innate-like" immune properties [6]. Interestingly, these B1 cells seem to have a predisposition to transform into hematologic malignancies, specifically CLL/SLL, mantle cell lymphoma, and marginal zone B-cell lymphoma [5,6]. As such, our patient meets the immunotype requirements of B-cell CLL as demonstrated by biopsy staining and flow cytometry. 

While further molecular investigations are not necessary for the diagnosis of CLL, additional testing provides prognostic value and helps guide treatment. As such, FISH analysis was performed and detected a chromosomal 13q14 deletion within our patient. In a pilot study by Dohner et al., deletion of the long arm on chromosome 13 (13q14) is the most common chromosomal abnormality found among CLL patients [7]. Within the deleted region of chromosome 13 is a portion of the *DLEU1* gene, the entire *DLEU2* gene, and the microRNA cluster miR-15a/16-1 [8]. It has been hypothesized that this specific microRNA cluster may act to regulate cell proliferation and apoptosis based on prior in vitro studies. In vivo studies of miR-15a/16-1 knock-out mice support this theory, which have demonstrated that this locus is particularly important in limiting proliferation of B cells by downregulating expression of “proliferation-associated proteins'' involved in the G0/G1 to S phase transition of the cell cycle [8]. This knockout mouse also demonstrated a CD5+ B-cell immunoproliferative phenotype, which continues to support the role of the miR-15a/16-1 cluster in the pathophysiology of CLL. Fortunately, of all the genetic aberrations associated with CLL (del11q, del13q, del17p, and trisomy 12q), the 13q14 deletion in our patient has the best prognosis and longest estimated survival time [7]. 

Although LC may commonly develop in B-cell CLL/SLL, it is rare to be the primary presenting symptom, as in our patient [1,4]. This presentation is often referred to as “aleukemic leukemia cutis” and is more often seen in patients diagnosed with AML rather than CLL/SLL [1]. While the underlying pathophysiology of LC remains largely unknown, current literature suggests that leukemia cells may infiltrate the blood-skin barrier in response to pre-existing antigens [4,9,10]. This may explain why LC has been known to arise in sites of infection, trauma, or foreign bodies. As such, Snorek et al. reported a case of relapsed B-cell CLL/SLL presenting as LC within the skin overlying an implantable cardioverter-defibrillator [11]. LC is also documented to arise without an inflammatory nidus. Fulton et al. described B-cell CLL/SLL with an initial presentation of LC in a pre-existing benign dermatologic lesion, which had been present for years [12]. This is notably different from our case, as our patient’s skin lesion appeared within one month. Maughan et al. presented a case of LC coexisting with dermatofibroma as the initial lesion in B-cell CLL/SLL [13]. This patient, like ours, denied lymphadenopathy and constitutional symptoms, and initial blood work was unremarkable. Both patients were diagnosed with B-cell CLL/SLL incidentally following biopsy of suspicious lesions.

## Conclusions

Leukemia patients commonly develop dermatologic manifestations, otherwise known as LC. However, it is not commonly the presenting symptom that leads to the diagnosis of leukemia. Our case describes a patient who presented with a primary concern of a new, evolving lesion without other systemic symptoms. A prompt skin biopsy revealed seborrheic keratosis in the epidermis and malignant lymphoid cells infiltrating into the dermis. After additional workup, the patient was diagnosed with B-cell CLL/SLL and referred to hematology. Fortunately for the patient, his leukemia demonstrated favorable genetic alterations, and he did not need to start immediate pharmacological treatment. As he has continued to follow up with his hematologist, he remains largely symptom-free and has not needed to start systemic treatment.
